# Breaking the Silence: A Case for Early Intervention in Profound Adolescent Hearing Loss

**DOI:** 10.7759/cureus.85569

**Published:** 2025-06-08

**Authors:** Alejandra C Vargas, Rommel F Portilla, Andrea G Ramirez, Isis X Muñoz, Melanie Caballero Garcia, Maria Guaman, Carlos Valladares

**Affiliations:** 1 Department of General Medicine, Chanchajalla Primary Health Care Facility, Ica, PER; 2 School of Medicine, Universidad de las Américas, Quito, ECU; 3 Facultad de Medicina Alberto Hurtado, Universidad Peruana Cayetano Heredia, Lima, PER; 4 Faculty of Medicine, National Autonomous University of Mexico, Mexico City, MEX; 5 Department of General Medicine, San Martín de Porres University, Lima, PER; 6 Faculty of Medicine, Universidad Católica Boliviana San Pablo, Santa Cruz de la Sierra, BOL; 7 Department of Internal Medicine, Community Medical Center, Toms River, USA

**Keywords:** adolescents, diagnostic limitations, post-viral hearing loss, progressive unilateral hearing loss, social withdrawal

## Abstract

Progressive unilateral hearing loss (PUHL) in adolescents is uncommon and presents diagnostic challenges due to its varied etiologies, including infections, ototoxicity, congenital anomalies, and autoimmune conditions, though many cases remain idiopathic. We report a 16-year-old male with a history of COVID-19, dengue at age 14, and recurrent headaches, who developed left-sided hearing loss progressing to profound unilateral deafness. Audiometry confirmed sensorineural hearing loss (SNHL) of 97 dB HL in the left ear and 35 dB HL in the right. Despite multiple evaluations, no definitive etiology was identified. Differential diagnoses included post-viral sequelae, congenital anomalies (e.g., Mondini dysplasia), and nasal obstruction, though insurance limitations restricted further testing. The patient discontinued hearing aid use due to limited benefit and experienced social withdrawal, impacting both academic performance and overall well-being. This case underscores the importance of early recognition and a systematic, multidisciplinary approach to PUHL, including imaging and genetic testing, given its potential impact on social development, mental health, and long-term outcomes.

## Introduction

Hearing loss affects more than 5% of the global population and can range from mild, temporary conditions, such as middle ear infections, to permanent impairments involving damage to the inner ear or auditory nerve. Profound sensorineural hearing loss (SNHL), defined as a hearing threshold exceeding 80 dB, can severely impact the ability to understand speech and perceive environmental sounds [1]. While hearing aids may provide some benefit, their effectiveness diminishes with increasing severity of loss. In such cases, cochlear implants, which bypass damaged structures to directly stimulate the auditory nerve, may offer improved auditory outcomes. Beyond hearing, profound auditory deprivation during critical developmental periods can also affect cognition, language acquisition, and social integration [2].

Although most research and clinical attention focus on bilateral hearing loss, unilateral hearing loss (UHL) represents a distinct and often overlooked challenge. Its causes are varied and include trauma, congenital anomalies, infections, autoimmune conditions, and vascular insults. In adolescents, UHL may progress slowly and present subtly, delaying detection until functional consequences, such as academic struggles or social withdrawal, become evident.

Progressive unilateral hearing loss (PUHL) in adolescents is particularly rare and diagnostically complex. Identifying the cause typically requires a multidisciplinary approach involving audiologic testing, neuroimaging, and targeted laboratory studies. However, access to these resources may be limited in under-resourced settings, constraining diagnostic accuracy and delaying appropriate intervention [3].

This case report presents an adolescent with PUHL of unclear origin in the context of limited access to diagnostic tools. The report aims not only to describe the clinical course but also to underscore the broader systemic challenges faced in the management of hearing loss in underserved populations. Recognizing these barriers is essential for improving early identification, guiding future resource allocation, and advocating for more equitable care.

## Case presentation

A 16-year-old male patient, previously healthy with no relevant personal or family medical history, presented with a two-year history of recurrent, episodic headaches of moderate intensity, without associated neurological symptoms. Over time, he developed progressive hearing loss in his left ear. The hearing loss was initially mild but gradually worsened, prompting medical consultation.

Initial audiometric and tympanometric evaluations were performed at a primary care outpatient clinic. Pure-tone audiometry revealed profound hearing loss in the left ear, with thresholds above 90 dB HL. The right ear showed mild hearing loss at 35 dB HL. The Pure Tone Average (PTA) was 97 dB HL in the left ear and 35 dB HL in the right. The Auditory Intelligibility Index (AII) indicated good speech discrimination in the right ear (83%) but significantly reduced discrimination in the left ear (67%).

Approximately one year later, follow-up audiometry showed normal hearing in the right ear, with a notch at 4 kHz suggestive of acoustic trauma, likely due to environmental noise exposure. The left ear continued to exhibit profound SNHL, indicating probable inner ear or central auditory pathway involvement. Tympanometry revealed a Type As curve in the right ear, suggestive of reduced ossicular or tympanic membrane mobility. No otoscopic or physical abnormalities were detected during examinations, apart from the hearing loss.

The patient was assessed at multiple medical centers, and the profound unilateral SNHL was confirmed. One evaluation suggested nasal turbinate hypertrophy as a potential contributing factor, although no imaging or endoscopic studies were performed to explore this further.

Other possible etiologies, such as post-infectious causes (e.g., COVID-19 or dengue, both prevalent in the region), were considered given the patient’s history of infection with both viruses around that time, COVID-19 confirmed by serologic testing. However, these were not further investigated due to limited access to diagnostic resources, including specific serologic testing or imaging (e.g., MRI). A congenital cause was also proposed, though no genetic testing or family screening was performed due to financial constraints. Furthermore, the lack of adequate documentation of the condition’s progression, particularly the absence of a regular timeline of hearing assessments, limits the ability to establish a clear onset or course. Supporting diagnostic tests, such as auditory brainstem response (ABR), balance assessments, or immunological studies, were also not performed, leaving potential neurological, immunological, or viral causes unconfirmed.

A hearing aid was prescribed for the left ear but provided limited benefit due to the severity of the hearing loss. No further audiological follow-up has been conducted in the past year, limiting the ability to assess current auditory function or disease progression. The patient now reports complete hearing loss in the left ear, with subjectively normal hearing in the right ear.

Psychosocially, he has expressed increased difficulty in school due to hearing challenges, including feelings of isolation and limited classroom participation. He also continues to experience recurrent headaches, without a clear pattern or associated neurological signs, which have persisted since the initial onset of symptoms.

## Discussion

This case is notable due to its insidious onset, lack of associated neurological symptoms, and the absence of a clear etiology despite multiple medical evaluations. Several potential causes have been considered, including post-viral complications, congenital anomalies, and nasal obstruction, yet no definitive diagnosis has been established. In this case, diagnostic challenges were amplified by limited resources, which delayed both diagnosis and treatment planning. This issue, particularly in vulnerable populations, can lead to delays in language development and communication problems, resulting in an avoidable and poorly managed disability. The limited access to diagnostic tools, such as MRI or serological testing, underscores a critical issue in healthcare disparities that can lead to delays in proper diagnosis and management. However, the auditory damage appears to be already established and unlikely to be reversible.

When considering possible causes of hearing loss, viral infections are among the primary etiologies, including arboviruses (dengue, Zika, and chikungunya) and COVID-19. These viruses may contribute to the development of SNHL through direct and indirect mechanisms. In SARS-CoV-2, it is known to damage hair cells or trigger immune-mediated responses that harm the cochlea. Furthermore, its propensity to cause microthrombosis and microhemorrhage may result in ischemic injury to the inner ear, which is extremely vulnerable due to its dependence on end-arterial blood supply [4]. In contrast to the patient's clinical presentation, characterized initially by recurrent headaches followed by progressive and irreversible hearing loss in the left ear, arboviral infections typically present with a sudden onset of hearing loss, often accompanied by vertigo and tinnitus [5]. The patient’s clinical evolution more closely resembles what is described in COVID-19. While the exact cause remains undefined, the most likely mechanisms include direct cochlear damage, microvascular alterations, and exacerbated immune responses. It is important to note that, due to limited available evidence, the only well-established finding is that young adults who contracted COVID-19 have an almost fourfold increased risk of developing hearing loss [6]. However, the absence of serological testing and complementary diagnostic studies limits the ability to determine the precise etiology of the hearing loss in this case.

Congenital inner ear malformations can remain asymptomatic until adolescence or early adulthood, becoming evident only after triggering events such as infections or trauma [7]. Examples include Mondini dysplasia, diagnosed in a patient following recurrent meningitis episodes, and enlarged vestibular aqueduct syndrome (EVA), identified in a 34-year-old patient with progressive hearing loss since childhood [8,9]. In comparison, the patient in question exhibits progressive and irreversible hearing loss without a clear triggering event. Although there is no evidence of a congenital malformation in this case, the underlying mechanism of endolymphatic dysfunction could share similarities with these conditions, highlighting the importance of imaging studies to rule out structural abnormalities.

Another potential cause considered was nasal obstruction, given its association with recurrent headaches and possible impact on middle ear function [10]. However, no confirmatory studies were performed, and the progressive, irreversible nature of the hearing loss makes it unlikely that this was the primary etiology. Nonetheless, nasal obstruction may have contributed to symptom exacerbation.

Moreover, the psychological aspect of this case is relevant, as it highlights the risk of misinterpreting symptoms and underscores the need for comprehensive audiological and neurological assessments to exclude organic causes before considering psychological explanations. After the diagnosis of hearing loss was confirmed, no psychological or educational interventions were initiated, despite the patient reporting difficulties in school, including feelings of isolation and limited classroom participation. This represents a missed opportunity to address the emotional and social consequences of untreated hearing loss. Future cases would benefit from integrated psychological support and academic accommodations to mitigate these long-term effects. Focusing on social reintegration, coping strategies, and educational accommodations is essential. Early counseling and intervention could have helped reduce the emotional distress and learning difficulties experienced by the patient.

This case highlights the need for detailed diagnostic evaluation in patients who develop progressive hearing loss, urging clinicians to consider not only the most common causes but also to adopt a broader, more comprehensive approach to identify less obvious underlying factors. Due to financial and infrastructural limitations, no definitive cause of the hearing loss could be established. If resources were available, further workup, including MRI, genetic testing, and serologic panels, would be crucial next steps in reaching an accurate diagnosis and guiding appropriate treatment. There is reason to believe that a guided diagnosis and well-planned treatment strategy could improve outcomes and reduce healthcare spending through more efficient care planning. The main challenge was the lack of diagnostic testing, largely due to financial limitations in the patient’s environment. As a result, no tests were performed to confirm or rule out the differential diagnoses based on clinical findings.

Lessons learned

This case illustrates the profound diagnostic and psychosocial impact of progressive UHL in adolescents, particularly in low-resource settings. It highlights the need for early, multidisciplinary evaluation, including audiological, neurological, and psychological assessments, even when advanced diagnostics are not immediately available. Misattributing symptoms to behavioral disorders without first excluding organic causes may delay critical interventions. Clinicians should maintain a high index of suspicion and prioritize early support strategies. Moreover, comprehensive care, including emotional, educational, and social support, should be initiated promptly after diagnosis to mitigate long-term functional and psychological consequences.

## Conclusions

This case highlights the diagnostic challenges of PUHL in resource-limited settings, where etiologies often remain unconfirmed. While imaging and genetic testing are ideal, early audiometric monitoring and psychosocial support are critical interim steps. Future cases should prioritize serological and structural evaluations when feasible.

Although rare, PUHL presents diagnostic difficulties due to its diverse etiologies, including anatomical, neurological, and genetic-hereditary causes, with clinical presentations ranging from acute to progressive loss. A comprehensive medical history and appropriate imaging studies are essential to establish the underlying cause. However, in this case, limited access to diagnostic tools hindered accurate diagnosis, underlining disparities in healthcare access and their impact on patient outcomes.

Equally important is the integration of mental health support, as adolescents with hearing loss are at increased risk of social isolation, depression, and anxiety. A multidisciplinary approach that addresses both medical and psychosocial aspects is essential to improving long-term outcomes.
